# FLAMS: Find Lysine Acylations and other Modification Sites

**DOI:** 10.1093/bioinformatics/btae005

**Published:** 2024-01-09

**Authors:** Hannelore Longin, Nand Broeckaert, Maarten Langen, Roshan Hari, Anna Kramarska, Kasper Oikarinen, Hanne Hendrix, Rob Lavigne, Vera van Noort

**Affiliations:** KU Leuven, Department of Microbial and Molecular Systems, Computational Systems Biology, Leuven 3001, Belgium; KU Leuven, Department of Biosystems, Laboratory of Gene Technology, Leuven 3001, Belgium; KU Leuven, Department of Microbial and Molecular Systems, Computational Systems Biology, Leuven 3001, Belgium; KU Leuven, Department of Biosystems, Laboratory of Gene Technology, Leuven 3001, Belgium; KU Leuven, Department of Microbial and Molecular Systems, Computational Systems Biology, Leuven 3001, Belgium; KU Leuven, Department of Microbial and Molecular Systems, Computational Systems Biology, Leuven 3001, Belgium; KU Leuven, Department of Microbial and Molecular Systems, Computational Systems Biology, Leuven 3001, Belgium; KU Leuven, Department of Microbial and Molecular Systems, Computational Systems Biology, Leuven 3001, Belgium; KU Leuven, Department of Biosystems, Laboratory of Gene Technology, Leuven 3001, Belgium; KU Leuven, Department of Biosystems, Laboratory of Gene Technology, Leuven 3001, Belgium; KU Leuven, Department of Microbial and Molecular Systems, Computational Systems Biology, Leuven 3001, Belgium; Leiden University, Institute of Biology Leiden (IBL), Leiden 2333 BE, The Netherlands

## Abstract

**Summary:**

Today, hundreds of post-translational modification (PTM) sites are routinely identified at once, but the comparison of new experimental datasets to already existing ones is hampered by the current inability to search most PTM databases at the protein residue level. We present FLAMS (Find Lysine Acylations and other Modification Sites), a Python3-based command line and web-tool that enables researchers to compare their PTM sites to the contents of the CPLM, the largest dedicated protein lysine modification database, and dbPTM, the most comprehensive general PTM database, at the residue level. FLAMS can be integrated into PTM analysis pipelines, allowing researchers to quickly assess the novelty and conservation of PTM sites across species in newly generated datasets, aiding in the functional assessment of sites and the prioritization of sites for further experimental characterization.

**Availability and implementation:**

FLAMS is implemented in Python3, and freely available under an MIT license. It can be found as a command line tool at https://github.com/hannelorelongin/FLAMS, pip and conda; and as a web service at https://www.biw.kuleuven.be/m2s/cmpg/research/CSB/tools/flams/.

## 1 Introduction

To expand the functional diversity accomplished by proteins, amino acids can be modified after protein synthesis by means of post-translational modifications (PTMs), which are commonly enzymatic in nature (Walsh *et al.* 2005). A wide range of PTMs exists, generally divided into three main categories: (i) proteolytic cleavage, (ii) linkage of amino acids, and (iii) (reversible) addition of chemical moieties. PTMs have been detected on all 20 of the standard amino acids (Li *et al.* 2022), and many proteins carry multiple PTMs simultaneously. By the modification of specific residues, PTMs influence the residues’ chemical properties, and as a result, PTMs can impact a protein’s charge, conformation and binding (e.g. Šoštarić and van Noort 2021), which could ultimately influence a protein’s function. This is referred to as the PTM code (Sims and Reinberg 2008).

dbPTM, one of the largest non-species-specific PTM databases (Ramazi and Zahiri 2021), contains 2 733 976 PTM sites, belonging to 76 categories, of which 1 921 021 in 72 categories are supported by experimental evidence (dbPTM version November 2023, Li *et al.* 2022). Among the most commonly modified residues are serine, with 1 209 458 PTM sites (mostly phosphorylation), and lysine with 724 627 reported PTM sites (mostly ubiquitination and acetylation). Phosphorylation has historically received the most attention, as reflected by the large amount of detected sites and the plethora of phosphorylation databases [a recent review summarized over 60 (Zhao *et al.* 2023)]. However, other post-translationally modified amino acids are coming to the fore, with lysine emerging as the amino acid capable of carrying the largest diversity of PTMs, as counted by dbPTM PTM categories. Nonetheless, resources for other amino acids are far more limited than for phosphorylation. For lysine, the second most represented amino acid in dbPTM, only one (non-species-specific) dedicated resource has been developed and maintained over the years: the Compendium of Protein Lysine Modifications (CPLM; http://cplm.biocuckoo.cn) (Liu *et al.* 2014). CPLM currently integrates and curates experimental protein lysine modification data from literature and 10 databases, and supplements it with rich metadata from 102 additional data sources (Zhang *et al.* 2022). CPLM stores information on 25 lysine modification types, encompassing the majority of lysine modification types.

As reported in dbPTM, hundreds of thousands of PTM sites have been discovered, driven by the continuously improving detection methods (Keenan *et al.* 2021). Due to the massive number of identified sites, functional assignments of these PTM sites are lagging behind. A comparison of the identified sites to those stored in PTM databases (by means of a BLASTp search for similar proteins in these databases) could aid in identifying already known, potentially even functionally assigned, modification sites. In addition, non-functionally assigned sites, especially those with repeated identifications across studies, could then be prioritized for functional characterization. Finally, the comparison to pre-existing datasets can also serve as a quality check, allowing researchers to quickly assess the overlap between their sites and those found during similar experiments. However, today, position-based searches, specifying both a PTM and its exact location, are generally not possible against major PTM databases. The major exception here appears to be the historically well researched PTM phosphorylation, with peptide matching in EPSD (Lin *et al.* 2021), and site searches in PhosphoSitePlus (Hornbeck *et al.* 2012, 2019). To address this issue, we developed FLAMS (Find Lysine Acylations and other Modification Sites), which serves to find previously identified modification sites in the same and similar proteins across species, by enabling a position-based search of the CPLM database and the experimentally supported subset of dbPTM. These databases were chosen to represent both an up-to-date, comprehensive overview of the PTM landscape (dbPTM) and add additional information on a large subset of these PTMs (CPLM), as lysine is, after serine (for which phosphorylation sites can already be searched for on a position-basis in other databases), the amino acid carrying the most PTM sites.

## 2 Implementation

**Figure 1.**
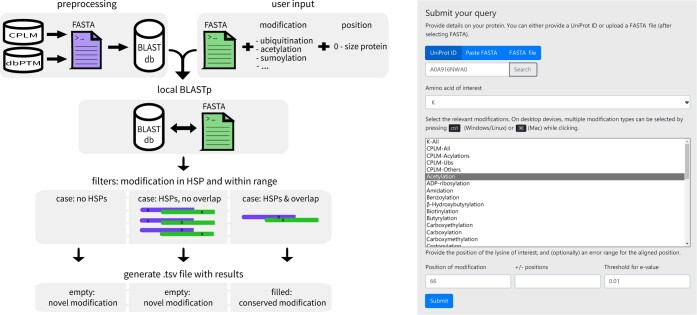
Left: Overview of the workflow of FLAMS in command line set-up. Briefly, the CPLM and dbPTM modification data is preprocessed into local BLAST databases (db), against which user provided queries are searched with BLASTp. High scoring pairs (HSPs) returned by BLAST are then filtered, to retain only those where modifications in CPLM align with the user requested modification. These hits are then returned to the user as a tab separated (.tsv) file. Right: Web interface for FLAMS.

FLAMS uses a sequence similarity-based approach to match a user provided query (consisting of at least a protein and a modification site) to similar proteins carrying the same PTM at a similar site. FLAMS does so by searching the CPLM and the experimental subset of the dbPTM for this information, and returning the results in a tabular format. It does this in a fast manner, by employing a clever trick: the data needed to identify conserved sites is stored directly in the FASTA headers, circumventing the need to consult any additional data source after the initial BLAST search. FLAMS can be used both from the command line and through its web interface. An overview of the workflow used by FLAMS is given below, and depicted visually in Fig. 1.

### 2.1 Preprocessing and aggregating protein PTM data

In the data aggregation and preprocessing stage, PTM data for a specific modification type are downloaded from the CPLM (Zhang *et al.* 2022) and/or the experimental part of dbPTM (Li *et al.* 2022). Each CPLM/dbPTM entry is converted into a FASTA formatted sequence record, storing relevant information in its header (i.e. the modification type, modification position, UniProt identifier, protein name, protein length and species name, as well as CPLM/dbPTM evidence). For dbPTM, this requires fetching the FASTA file of each protein through UniProt’s API (The UniProt Consortium 2023), as sequence information is not included in the dbPTM download files. All entries are written to one multi-FASTA file per data source (i.e. one for CPLM and one for dbPTM) using BioPython v.1.79 SeqIO (Cock *et al.* 2009). As dbPTM entries depend on the downloaded UniProt FASTA file, it is possible that dbPTM entries are excluded from the multi-FASTA file, e.g. when the UniProt identifier in dbPTM is obsolete. The CPLM and dbPTM multi-FASTA files are then integrated into one multi-FASTA file per modification type. These FASTA files are pre-generated, and hosted on Zenodo (doi: 10.5281/zenodo.10143463). FASTA files will be updated in accordance to dbPTM and/or CPLM updates.

### 2.2 Command line interface

#### 2.2.1 Creating local BLAST modification databases

The multi-FASTA file containing all CPLM and/or dbPTM entries for a specific modification type is subsequently downloaded from Zenodo and used to generate a local BLAST database with BLAST+ v.13 makeblastdb (Camacho *et al.* 2009). This procedure is invoked once per modification type, namely the first time this specific modification type is called by the application.

#### 2.2.2 Assessing validity of user-provided arguments

Before actually performing a search for known PTM sites, user-provided arguments are parsed and checked for validity. Most relevantly, FLAMS checks (i) if the provided FASTA file is recognized as such by SeqIO, (ii) if the specified modification position is within the range of the protein size, and (iii) if the specified modification position points to an amino acid that is capable of carrying the given modification type(s). If users provide a UniProt identifier instead of a local FASTA file, FLAMS downloads the corresponding FASTA file through UniProt’s API (The UniProt Consortium 2023).

#### 2.2.3 Detecting conserved protein modifications

If all arguments are valid, FLAMS performs a BLASTp search against the local BLAST database(s) containing the PTM data for the specified modification type(s). High scoring pairs (HSPs) are filtered in three stages. First, only HSPs with an e-value ≤ the user-specified e-value are retained. Second, only HSPs containing a modified amino acid in both the aligned query and target sequence are retained. Finally, only HSPs where the queried modification site aligns (within the user-specified range) to a modified amino acid in the target sequence, are retained.

For each retained HSP, a row is written to the output file in .tsv format. Each row contains information on one conserved PTM, and specifies information on (i) the protein (UniProt identifier and protein name), (ii) the modification (type, position and the sequence surrounding this modification), (iii) the species, (iv) the BLASTp run (E-value, identity and coverage), (v) CPLM hits, if any (CPLM ID, evidence code and evidence links), and (vi) dbPTM hits, if any (evidence code and links).

#### 2.2.4 Batch mode

In batch mode, FLAMS first performs additional checks on the batch input file, verifying the provided UniProt identifiers and positions. Then, FLAMS is run iteratively (going through described stages 1–3), taking the UniProt identifiers and positions specified in the batch file as input and creating one output file per line in the batch file. Any additional specified command line options will be applied to all runs in the batch.

### 2.3 Web tool

A modified version of the command line tool is made available as a web interface. The web interface is created from the raw Python3 scripts with Flask v.2.2.2. A Docker image, combining the Flask application with Gunicorn v.20.1.0, is created, and hosted on the KU Leuven hosting platform Elsschot.

## 3 Usage

The goal of FLAMS is to provide users a straightforward way to assess whether their modification sites have been reported previously, either exactly as found, or in similar proteins (as examined with a BLASTp search against dbPTM and CPLM). To showcase how this can be done, we examined whether the TatA acetylation on K66 in *Dehalococcoide mccartyi* strain CBDB1, as described by Greiner-Haas *et al.* (2021), had been previously detected. Using the command ‘FLAMS—id A0A916NWA0 -p 66 -m acetylation -o tatA.tsv’, we can find that this exact acetylation site has been previously reported in *Escherichia coli* (Weinert *et al.* 2013). In a more extensive example use case, we assessed all 152 modifications of the acylproteome of *Syntrophus aciditrophicus* reported by Muroski *et al.* (2022) for their novelty, and found that it contains 72 modifications not yet present in the CPLM and dbPTM database and 80 that were previously reported. Data preprocessing and the FLAMS utilities shows that most of these are only conserved in a few other species, some sites are highly conserved. Details and more examples can be found in the iPython notebook (Supplementary Material).

## 4 Discussion

Today, PTM studies frequently report a large number of identified sites, often varying between a hundred and over a thousand sites, depending on the modification type, equipment, protocols, etc Typically, the number of identified sites vastly eclipses the number of sites that can be investigated experimentally, and researchers attempt to explain the majority of the identified sites by (i) protein set enrichment analysis and (ii) comparison to other PTM studies. However, these comparisons are usually limited to a small set of other modification datasets, carefully selected by the authors. For example, for acetylation, one of the most studied lysine modification types, cross-study comparisons are often limited to less than 10 datasets, as authors have to compare the different datasets manually following a BLAST between the different acetylomes (e.g. Chen *et al.* 2017, Pang *et al.* 2020, Sun *et al.* 2018). As a result, some authors report only protein-level conservation of acetylation, instead of a more informative and precise residue position-based conservation (e.g. Chen *et al.* 2017, Meng *et al.* 2016).

With FLAMS, it becomes straightforward to carry out the task of identifying previously reported modification sites stored in dbPTM and CPLM. Briefly, the example cases (Supplementary Material) showed that FLAMS can be used (i) to quickly verify whether modifications in a specific protein have been reported previously, (ii) to assess whether findings in one species might translate to other species, and (iii) to systematically assess the novelty and conservation of reported modification sites. The key to FLAMS’ success is the clever use of FASTA headers, where all relevant search information is concisely stored. This innovative approach may also help other future projects where residue information is needed.

To conclude, FLAMS facilitates the comparison of new PTM datasets to currently known ones, by allowing a position-based search against the contents of the entire CPLM database and the experimentally supported subset of dbPTM. As such, it automates the oftentimes time-consuming manual comparisons and eliminates potential selection bias, stemming from the limited number of datasets used in these comparisons. Due to FLAMS’ implementation as a Python3 command line tool, FLAMS can readily be integrated into larger analysis pipelines, which will likely become increasingly important in the field of PTMs, where the amount of data is increasing faster than the functional interpretation thereof. Its intuitive web interface also guarantees smooth access to the tool for the many experimentalists in the field.

## Supplementary Material

btae005_Supplementary_Data

## References

[btae005-B1] Camacho C , Coulouris G, Avagyan V et al BLAST+: architecture and applications. BMC Bioinformatics 2009;10:421. 20003500 10.1186/1471-2105-10-421 PMC2803857

[btae005-B2] Chen Z , Zhang G, Yang M et al Lysine acetylome analysis reveals photosystem II manganese-stabilizing protein acetylation is involved in negative regulation of oxygen evolution in model Cyanobacterium *Synechococcus* sp. PCC 7002. Mol Cell Proteomics 2017;16:1297–311. 28550166 10.1074/mcp.M117.067835 PMC5500762

[btae005-B3] Cock PJA , Antao T, Chang JT et al Biopython: freely available Python tools for computational molecular biology and bioinformatics. Bioinformatics 2009;25:1422–3. 19304878 10.1093/bioinformatics/btp163 PMC2682512

[btae005-B22] Greiner-Haas F , Bergen MV, Sawers G et al Changes of the proteome and acetylome during transition into the stationary phase in the organohalide-respiring dehalococcoides mccartyi strain CBDB1. Microorganisms 2021;9:365. 10.3390/microorganisms9020365. 33673241 PMC7918482

[btae005-B4] Hornbeck PV , Kornhauser JM, Latham V et al 15 years of PhosphoSitePlus^®^: integrating post-translationally modified sites, disease variants and isoforms. Nucleic Acids Res 2019;47:D433–41. 30445427 10.1093/nar/gky1159 PMC6324072

[btae005-B5] Hornbeck PV , Kornhauser JM, Tkachev S et al PhosphoSitePlus: a comprehensive resource for investigating the structure and function of experimentally determined post-translational modifications in man and mouse. Nucleic Acids Res 2012;40:D261–70. 22135298 10.1093/nar/gkr1122 PMC3245126

[btae005-B6] Keenan EK , Zachman DK, Hirschey MD et al Discovering the landscape of protein modifications. Mol Cell 2021;81:1868–78. 33798408 10.1016/j.molcel.2021.03.015 PMC8106652

[btae005-B7] Li Z , Li S, Luo M et al dbPTM in 2022: an updated database for exploring regulatory networks and functional associations of protein post-translational modifications. Nucleic Acids Res 2022;50:D471–9. 34788852 10.1093/nar/gkab1017 PMC8728263

[btae005-B8] Lin S , Wang C, Zhou J et al EPSD: a well-annotated data resource of protein phosphorylation sites in eukaryotes. Brief Bioinform 2021;22:298–307. 32008039 10.1093/bib/bbz169

[btae005-B9] Liu Z , Wang Y, Gao T et al CPLM: a database of protein lysine modifications. Nucleic Acids Res 2014;42:D531–6. 24214993 10.1093/nar/gkt1093 PMC3964993

[btae005-B10] Meng Q , Liu P, Wang J et al Systematic analysis of the lysine acetylome of the pathogenic bacterium *Spiroplasma eriocheiris* reveals acetylated proteins related to metabolism and helical structure. J Proteomics 2016;148:159–69. 27498276 10.1016/j.jprot.2016.08.001

[btae005-B21] Muroski JM , Fu JY, Nguyen HH et al The acyl-proteome of syntrophus aciditrophicus reveals metabolic relationships in benzoate degradation. Mol Cell Proteomics 2022;21:100215. 10.1016/j.mcpro.2022.100215. 35189333 PMC8942843

[btae005-B11] Pang H , Li W, Zhang W et al Acetylome profiling of *Vibrio alginolyticus* reveals its role in bacterial virulence. J Proteomics 2020;211:103543. 31669173 10.1016/j.jprot.2019.103543

[btae005-B12] Ramazi S , Zahiri J. Post-translational modifications in proteins: resources, tools and prediction methods. Database 2021;2021:baab012. 33826699 10.1093/database/baab012 PMC8040245

[btae005-B13] Sims RJ , Reinberg D. Is there a code embedded in proteins that is based on post-translational modifications? Nat Rev Mol Cell Biol 2008;9:815–20. 18784729 10.1038/nrm2502

[btae005-B14] Šoštarić N , van Noort V. Molecular dynamics shows complex interplay and long-range effects of post-translational modifications in yeast protein interactions. PLoS Comput Biol 2021;17:e1008988. 33979327 10.1371/journal.pcbi.1008988 PMC8143416

[btae005-B15] Sun X-L , Yang Y-H, Zhu L et al The lysine acetylome of the nematocidal bacterium bacillus nematocida and impact of nematode on the acetylome. J Proteomics 2018;177:31–9. 29425737 10.1016/j.jprot.2018.02.005

[btae005-B16] The UniProt Consortium. UniProt: the universal protein knowledgebase in 2023. Nucleic Acids Res 2023;51:D523–31. 36408920 10.1093/nar/gkac1052 PMC9825514

[btae005-B17] Walsh CT , Garneau-Tsodikova S, Gatto GJ et al Protein posttranslational modifications: the chemistry of proteome diversifications. Angew Chem Int Ed Engl 2005;44:7342–72. 16267872 10.1002/anie.200501023

[btae005-B18] Weinert BT , Schölz C, Wagner SA et al Lysine succinylation is a frequently occurring modification in prokaryotes and eukaryotes and extensively overlaps with acetylation. Cell Rep 2013;4:842–51. 23954790 10.1016/j.celrep.2013.07.024

[btae005-B19] Zhang W , Tan X, Lin S et al CPLM 4.0: an updated database with rich annotations for protein lysine modifications. Nucleic Acids Res 2022;50:D451–9. 34581824 10.1093/nar/gkab849 PMC8728254

[btae005-B20] Zhao M-X , Chen Q, Li F et al Protein phosphorylation database and prediction tools. Brief Bioinform 2023;24:bbad090. 36896955 10.1093/bib/bbad090

